# Advances in antisense oligonucleotide treatment for cancer

**DOI:** 10.1093/jjco/hyag017

**Published:** 2026-02-03

**Authors:** Lu Zhu, Pariha Muhtar, Susumu Goyama, Akihide Yoshimi

**Affiliations:** Division of Cancer RNA Research, National Cancer Center Research Institute, 5-1-1 Tsukiji, Chuo-ku, Tokyo 104-0045, Japan; Department of Hematology and Oncology, Graduate School of Medicine, The University of Tokyo, 7-3-1, Hongo, Bunkyo-ku, Tokyo 113-0033, Japan; Division of Cancer RNA Research, National Cancer Center Research Institute, 5-1-1 Tsukiji, Chuo-ku, Tokyo 104-0045, Japan; Department of Hematology and Oncology, Graduate School of Medicine, The University of Tokyo, 7-3-1, Hongo, Bunkyo-ku, Tokyo 113-0033, Japan; Department of Cancer Cellular Signaling, Graduate School of Medicine, The University of Tokyo, 7-3-1, Hongo, Bunkyo-ku, Tokyo 113-0033, Japan; Department of Hematology and Oncology, Graduate School of Medicine, The University of Tokyo, 7-3-1, Hongo, Bunkyo-ku, Tokyo 113-0033, Japan; Department of Computational Biology and Medical Sciences, Graduate School of Frontier Sciences, The University of Tokyo, 4-6-1 Shirokanedai, Minato-ku, Tokyo 108-8639, Japan; Division of Cancer RNA Research, National Cancer Center Research Institute, 5-1-1 Tsukiji, Chuo-ku, Tokyo 104-0045, Japan; Department of Cancer Cellular Signaling, Graduate School of Medicine, The University of Tokyo, 7-3-1, Hongo, Bunkyo-ku, Tokyo 113-0033, Japan

**Keywords:** molecular targeted therapy, oligonucleotides, antisense, RNA splicing, neoplasms, clinical trial

## Abstract

RNA therapeutics, including antisense oligonucleotides (ASOs), have emerged as a promising class of drugs, with several already approved for clinical use. To date, most approved ASO-based RNA therapies target non-malignant disorders such as neurodegenerative diseases, and only a single therapy in this class has been approved for cancer. Notably, nearly half of existing RNA therapeutics act by modulating RNA splicing. Given the growing evidence implicating aberrant RNA splicing in cancer pathogenesis, the development of ASO-based therapeutics for oncologic indications is expected to accelerate. More than 250 clinical trials have evaluated oligonucleotide agents targeting diverse cancer-associated molecules, with several showing encouraging early results. In this review, we summarize recent advances in understanding cancer biology relevant to ASO-based therapies and highlight ongoing progress in the development of RNA-targeted approaches for cancer treatment.

## Teaser

**Mini-abstract** RNA therapeutics are rapidly expanding into oncology. With growing evidence linking aberrant RNA processing to cancer and increasing clinical progress, RNA-targeted strategies show emerging promise for future cancer treatment.

## Introduction

In recent years, various RNA species have emerged as promising therapeutic agents, including antisense oligonucleotides (ASOs), messenger RNA (mRNA), small interfering RNA (siRNA), microRNAs (miRNA), and RNA aptamers. The rapid development and global deployment of mRNA vaccines during the COVID-19 pandemic demonstrated that RNA-based therapeutics can be safely and effectively applied in clinical practice. This success generated significant scientific, medical, and societal impact and highlighted the therapeutic potential of RNA medicines in real-world settings.

Building on the demonstrated success of RNA-based therapeutics, one of the earliest strategies developed for therapeutic RNA manipulation was the ASO technology. Over decades of development, the continued optimization of oligonucleotide chemistry has enhanced the stability, target affinity, and cellular delivery of ASOs, supporting their use in both experimental research and clinical applications. To date, ASOs constitute the largest class of approved RNA-based drugs. Multiple ASOs have been approved for the treatment of diseases across different tissues, such as endocrine, cardiovascular, and neurological/neuromuscular systems [1]. While most FDA-approved ASOs target rare genetic diseases, increasing evidence indicates that ASOs may also hold great potential in cancer therapy. Therefore, in this review, we provide a concise summary of the recent advances in ASO-based RNA therapeutics for cancer.

## The development and application of ASOs

Since the concept of ASOs was first proposed in 1978 [2], nearly 50 years of continuous development have transformed ASOs from a theoretical idea into a clinically validated therapeutic platform. Continuous advancements in chemical structure modifications, the discovery of mechanisms of action, improvements in delivery strategies and dosing strategies, and safety optimization have progressively expanded the clinical applicability of ASOs. The approval of multiple ASO drugs by the FDA and European Medicines Agency (EMA), and positive clinical outcomes across a wide range of diseases highlight the unique potential of ASOs in RNA therapeutics [3]. Importantly, by enabling once considered an “undruggable” RNA to become a viable therapeutic target, ASO technology has opened new possibilities for precision and personalized medicine.

### The mechanisms of action of ASOs

**Figure 1:**
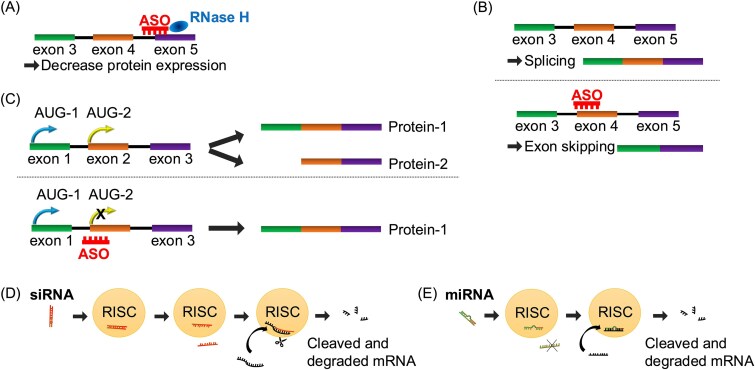
Schematic representation for mechanisms of actions of oligonucleotide therapeutics. (A) When antisense oligonucleotides (ASOs) bind to the target mRNAs, RNase H recognizes the RNA–DNA heteroduplex and degrades the mRNAs. (B) ASOs sterically block the binding of splicing factors or other RNA binding protein to *cis*-element and modulate splicing. (C) Oligonucleotides can also regulate translational initiation. (D) siRNAs cause RNA-induced silencing complex (RISC)-mediated degradation of mRNA targets. (E) miRNAs mediate post-transcriptional gene regulation through incorporation into the RISC, leading to mRNA degradation or translational repression.

In general, ASOs are small (~18–30 nucleotides), single-stranded nucleic acid polymers that are complementary to the specific RNA through Watson–Crick base-pairing [4]. The main mechanisms of approved and clinically developed ASOs are roughly classified into the following two categories (Fig. 1A–B) [3, 5].

#### Occupancy-mediated degradation

Ribonuclease H1(RNase H1)–dependent degradation represents the most clearly understood route of ASO-mediated gene silencing [6]. In this way, ASOs hybridize to their target mRNA to form an RNA–DNA heteroduplex that is specifically recognized and cleaved by RNase H1 endonuclease, leading to mRNA degradation and downregulation of the target gene expression (Fig. 1A). This mechanism is also commonly exploited by currently approved ASOs. Recent studies propose that ASOs can be designed to induce Argonaute 2–RNA-Induced Silencing Complex (Ago2-RISC) mediated cleavage by forming RNA–RNA duplexes with their target transcripts [7]. However, this concept remains at the proof-of-principle stage and has not yet been translated into therapeutic ASOs. Moreover, ASOs may also reduce target RNA levels through enzyme-independent RNA decay pathways, including nonsense-mediated decay (NMD) [8, 9] and no-go decay (NGD) [10].

#### Occupancy-only mechanisms

Splice modulation is one of the most major purposes in this category. ASOs modulate pre-mRNA splicing by sterically blocking the interaction of splicing factors with *cis*-acting regulatory elements such as splice sites, exonic splicing enhancers (ESEs), or intronic splicing silencers (ISSs). This mode of action enables several currently approved ASO drugs to redirect exon usage—either promoting exon skipping or exon inclusion, ultimately restoring the production of a functional protein and achieving therapeutic benefit (Fig. 1B) [11–13]. ASOs can also act through steric hindrance, binding to mRNA and blocking or inhibiting its translation, thereby reducing protein expression (Fig. 1C) [14].

### The generation of ASO

ASOs are inherently susceptible to nuclease-mediated degradation in their unmodified (naked) form, resulting in a short plasma half-life of <5 min in peripheral circulation [15, 16]. In addition, their phosphodiester backbone limits cellular uptake, as only a small fraction of ASOs can escape from endomembrane compartments. Furthermore, ASOs often exhibit poor biodistribution and pharmacokinetic behavior, along with suboptimal binding affinity to complementary RNA sequences [17]. To overcome these limitations, extensive efforts have focused on chemical modifications of ASOs, together with conjugation and delivery strategies, and other emerging optimization methods. Consequently, three successive generations of modified ASOs have been developed, which can be categorized as follows. The key features of each generation are summarized in Table 1.

**Table 1:** Overview of antisense oligonucleotide (ASO) generations and design strategies

Category/generation	Key chemical features	Mechanism of action	Advantages	Limitations/notes
First-generation ASOs	Phosphorothioate (PS) backbone	RNase H–mediated target RNA degradation	Improved nuclease resistance; enables target RNA cleavage	Lower binding affinity; off-target effects and toxicity
Second-generation ASOs	2′-Ribose modifications (2′-O-Me, 2′-MOE, 2′-F)	Primarily steric blocking or splicing modulation; limited RNase H activity	Higher target affinity and stability than first generation	Sequence-dependent hepatotoxicity and potential nephrotoxicity; nuclear delivery; tumor heterogeneity; off-target effects
Third-generation ASOs	Advanced structural modifications (LNA, PMO, PNA)	Steric blocking, splicing regulation, or translation inhibition	Very high affinity, stability, and improved pharmacokinetics	Limited RNase H compatibility; poor cellular uptake; dose-limiting toxicity; delivery dependence
Delivery-based strategies				
Bioconjugate-based approaches	GalNAc, antibody (AOC), lipid, peptide, aptamer	Receptor-mediated uptake	Cell- or tissue-specific delivery	Mostly liver based
Carrier-based approaches	Lipid nanoparticles, polymeric carriers, exosomes	Endocytosis-mediated delivery	Expanded extrahepatic tissue targeting	Endosomal escape

#### First-generation ASO

In first-generation ASOs, the phosphate backbone is chemically modified to improve stability. In these ASOs, the non-bridging oxygen atom is replaced by sulfur (phosphorothioate, PS ASOs), a methyl group (methyl-phosphonate ASOs), or an amine group to generate N3′–P5′ phosphoramidate ASOs. These modifications primarily increase resistance to nuclease-mediated degradation [16]. In addition, increased binding affinity to plasma proteins contributes to prolonged circulation time and improved pharmacokinetic stability [18–20]. The most widely used PS backbone modification dramatically prolongs the half-life from minutes to days. This property also helps the first-generation ASOs bind to intracellular proteins and improve enrichment in the cytoplasm as well as in the nucleus [21].

Another advantage of these modifications in the first-generation ASOs is their capability to enhance RNase H activity. Therefore, RNase H recognizes the ASO/mRNA complex and degrades the target mRNA within the complex (Fig. 1A) [22, 23].

Fomivirsen, the first approved ASO by the FDA in 1998 and the EMA in 1999, was a first-generation ASO with the PS backbone which targets the major immediate early region (IE2) proteins of cytomegalovirus (CMV), a critical element to produce essential viral proteins. To evaluate the efficacy of fomivirsen for newly diagnosed CMV retinitis in patients with acquired immunodeficiency syndrome (AIDS), a multicenter, prospective clinical trial was performed by the Vitravene Study Group [23, 24]. In this study, the authors concluded that fomivirsen is an effective treatment for CMV treatment in patients with AIDS with an acceptable safety profile. Although the efficacy of fomivirsen seemed excellent, this drug was withdrawn from use owing to limited clinical need [25]. Due to their relatively low hybridization specificity and undesired protein interactions that may cause off-target effects, PS-based first-generation ASOs have been largely phased out from current development programs [26, 27].

#### Second-generation ASO

Second-generation ASOs were designed to enhance target specificity, molecular stability, and safety compared to first-generation compounds, enabling more effective therapeutic applications [28–30]. In second-generation ASOs, the 2′ position of the ribose is chemically modified. More specifically, the hydroxyl group (–OH) at the 2′ end of the ribose is replaced with small substituents such as a methyl, a methoxyethyl, or a fluoro group, resulting in the formation of 2′-*O*-methyl (2′-OMe), 2′-*O*-methoxyethyl (2′-O-MOE), and 2′-fluoro (2′-F) modifications, respectively [30–33]. In general, second-generation ASOs exhibit higher nuclease resistance and stronger target affinity than first-generation compounds. However, their increased chemical stability may introduce new types of toxicity in certain contexts. A key distinction between first- and second-generation ASOs is that many of the latter, especially those fully modified at the 2′ position, do not induce RNase H–mediated degradation [34]. Consequently, fully modified second-generation ASOs are generally used as steric-blocking oligonucleotides or splicing modulators, rather than as RNA-degrading agents (Fig. 1B–C) [21].

A representative example of the second-generation ASO is Nusinersen (Spinraza®), an ASO that was specifically designed for the treatment of spinal muscular atrophy (SMA) [35, 36]. Nusinersen is a fully PS- and 2′-O-MOE–modified *steric-blocking ASO* that modulates pre-mRNA splicing rather than inducing RNase H–mediated degradation. SMA is an autosomal recessive disease that affects 1:7000–11 000 live births [37], characterized by the progressive loss of motor neurons, muscular atrophy, and high mortality in infants. In most cases, homozygous deletions or mutations in the *Survival Motor Neuron 1 (SMN1)* gene result in loss of SMN protein. Although SMN protein is encoded by nearly identical *SMN1* and *SMN2* genes, exon 7 of *SMN2* is predominantly skipped in most tissues [38], generating unstable SMN protein. When the *SMN1* gene is genetically lost, only a small amount of functional SMN protein is produced because the *SMN2* gene cannot fully compensate due to the exon 7 skipping. Nusinersen prevents the exon 7 skipping of SMN2 by blocking an ISS called ISS-N1, which spans from the 10th to 24th positions of intron 7 and normally suppresses exon 7 inclusion. Clinical trials demonstrated that Nusinersen improves the symptoms and survival of infantile- and childhood-onset SMA patients [39–43], leading to the FDA approval in 2016 and the EMA approval in 2017.

Another example is Milasen (approved in 2018), which shares similar PS and 2′-O-MOE chemical features with Nusinersen. Milasen corrects a patient-specific splicing defect in the major facilitator superfamily domain-containing protein 8 (*MFSD8*) gene—the first “n-of-1” personalized ASO therapy ever approved [13, 44].

#### Third-generation ASO

In pursuit of enhanced RNA binding affinity, chemical stability, and favorable pharmacokinetic profiles, third-generation ASOs adopt more complex structural modifications than their first- and second-generation predecessors [45].

One of the most widely used chemical modifications in third-generation ASOs is the locked nucleic acid (LNA), which introduces a 2′,4′-methylene bridge linking the 2′-oxygen and 4′-carbon of the ribose. This constraint locks thereby markedly enhancing hybridization strength and raising the melting temperature [46]. On the other hand, LNA-modified ASOs generally do not activate RNase H–mediated cleavage and instead act through a steric-blocking mechanism [47, 48]. However, extensive LNA incorporation can lead to hepatotoxicity. To overcome this, 2′-constrained ethyl (2′-cEt) nucleosides were developed as a more flexible bridged analog. ASOs containing 2′-cEt exhibit LNA-like potency and nuclease resistance but with improved safety and pharmacokinetic profiles [49, 50].

Another important subclass of third-generation ASOs is the phosphorodiamidate morpholino oligomer (PMO), which is frequently used to block mRNA translation and modulate splicing [51]. In PMOs, the backbone is linked by phosphorodiamidates instead of phosphates, and the ribofuranose rings are replaced by morpholino rings. The PMO structure is uncharged at physiological pH, which results in low plasma protein binding. This neutral backbone enhances molecular stability and biodistribution, allowing PMOs to exert antisense effects primarily through steric blocking and splicing modulation. However, due to their limited cellular uptake and rapid systemic clearance, relatively high doses are required to achieve therapeutic efficacy [52, 53]. At the same time, the inter-nucleotide phosphorodiamidate linkage and the absence of charged carboxyl groups give PMOs strong resistance to nucleases, proteases, and esterases, while maintaining their high affinity for complementary RNA. This stability enables PMOs to form durable duplexes with target mRNAs, thereby effectively interfering with splicing and translation [54]. Several PMO-based drugs have been approved by the FDA for the treatment of Duchenne muscular dystrophy (DMD), including eteplirsen (approved in 2016)**,** golodirsen (approved in 2019), viltolarsen (approved in 2020), and casimersen (approved in 2021). These drugs modulate pre-mRNA splicing by sterically blocking splice sites to induce exon skipping, restoring the reading frame and allowing translation of a partially functional dystrophin protein in DMD [55–58].

The third subclass of ASOs in this category is the peptide nucleic acids (PNAs), in which the ribose-phosphate backbone is substituted with a polyamide backbone [59, 60]. This unnatural backbone and neutral charge of PNAs confer resistance to degradation and a strong affinity with their target mRNAs. On the other hand, decreased cellular uptake, limited water solubility, and rapid clearance via renal pathway are the main challenges [21, 53].

### Delivery-based strategies for ASOs

Although most clinically approved ASOs have historically been administered as *naked oligonucleotides* relying solely on backbone and sugar modifications for stability and passive tissue distribution [61], a clear design shift has recently emerged in oligonucleotide therapeutics. This new trend is driven in part by the success of *N*-acetylgalactosamine (GalNAc)-conjugated siRNA drugs including givosiran, lumasiran, inclisiran, vutrisiran, nedosiran, and fitusiran approved between 2019 and 2025 [62, 63], which target a variety of diseases across systems including metabolic, hepatic, and hematological disorders. These siRNA drugs use hepatocyte-targeted GalNAc delivery to achieve gene silencing, resulting in clinically meaningful efficacy and an overall favorable safety profile [64–66].

Inspired by these advances, several GalNAc-conjugated ASOs have recently been approved by the FDA since 2023, marking a shift from relying on chemical optimization to a new design approach that combines molecular modification with active, ligand-guided delivery [67].

Eplontersen is the first GalNAc-conjugated ASO approved by the FDA in 2023 [68]. It is a GalNAc-2′-MOE gapmer ASO that targets transthyretin (TTR) mRNA in the liver, thereby reducing circulating TTR protein levels and amyloid deposition [69]. This mechanism provides therapeutic benefit in patients with transthyretin amyloidosis (ATTR). In patients with ATTR polyneuropathy, eplontersen lowered serum TTR levels, decreased neuropathy impairment, and improved health-related quality of life [70].

Olezarsen is a GalNAc-conjugated 2′-MOE ASO targeting apolipoprotein C-III (ApoC-III) mRNA [71]. It was approved by the FDA in 2024 for the treatment of familial chylomicronemia syndrome (FCS) [72]. By binding to ApoC-III mRNA, olezarsen promotes RNase H1–mediated degradation of the transcript, leading to a reduction of serum ApoC-III protein and subsequently increased clearance of plasma triglycerides (TG) and very-low-density lipoproteins (VLDL) [71]. In recent Phase III clinical trials, olezarsen treatment produced significantly greater reductions in serum TG levels at 6 months than placebo among patients with moderate hypertriglyceridemia and elevated cardiovascular risk. Moreover, in patients with FCS, olezarsen represents a promising therapeutic option for effectively lowering plasma triglyceride levels [73, 74].

Donidalorsen, approved in August 2025, is also a GalNAc-conjugated 2′-MOE ASO for the prevention of hereditary angioedema (HAE) attacks [75]. It binds to *prekallikrein (PKK) mRNA* and induces RNase H1–mediated degradation, thereby reducing the production of PKK protein [76]. In Phase III clinical trials, donidalorsen treatment significantly reduced the rate of hereditary angioedema attacks compared with placebo [77].

Since GalNAc conjugation primarily enables liver-targeted delivery, developing strategies for extrahepatic delivery has become a major focus in next-generation oligonucleotide therapeutics. Antibody–oligonucleotide conjugates (AOCs) have emerged as a promising solution. By combining the antibody’s targeting ability with the sequence specificity of the oligonucleotide, AOCs enable precise delivery of oligonucleotides to specific cells or tissues [78, 79]. A leading example of the AOC ASO is DYNE-101, which targets dystrophia myotonica protein kinase (DMPK) mRNA in skeletal muscle via human transferrin receptor 1–mediated delivery. Preclinical studies demonstrated correction of spliceopathy and reduction of DMPK RNA and nuclear foci in multiple myotonic dystrophy type 1 models. The compound is now in Phase I/II clinical evaluation (NCT05481879) [80].

The conjugation-based delivery systems discussed above belong to bioconjugate-based approaches. Other types of bioconjugates currently under development include lipid conjugates, aptamer conjugates, and peptide conjugates, each aiming to enhance the specificity and efficiency of oligonucleotide delivery to target tissues. In addition to conjugate-based designs, another major class of delivery platforms is carrier-based approaches, which include lipid-based nanocarriers, polymeric nanocarriers, exosomes, and cell-penetrating peptide–based formulations. These systems complex oligonucleotides to protect them from degradation and facilitate cellular uptake, representing complementary strategies to expand tissue targeting beyond the liver [79].

## RNA therapeutics targeting cancers

Precision oncology has become an important direction in cancer treatment, as targeted therapies have significantly improved cancer management by selectively inhibiting genetic alterations and their molecular consequences that are essential for tumor development. Successful examples of targeted therapies include tyrosine kinase inhibitors for Philadelphia chromosome–positive chronic myeloid leukemia (CML) [81] and acute lymphoblastic leukemia (ALL) [82], vemurafenib for BRAF V600E–mutant metastatic melanoma [83], anti-epidermal growth factor receptor (EGFR) antibodies for metastatic colorectal cancer [84], and anti-human epidermal growth factor receptor 2 (HER2) antibodies for HER2-positive breast cancer [85].

Despite extensive efforts, many “undruggable” genetic alterations still cannot be targeted using traditional approaches that primarily target proteins. Advances in RNA biology and transcriptomics have deepened our understanding of tumorigenesis. RNA therapeutics enable direct, selective regulation of gene expression at the post-transcriptional level, allowing more precise intervention in tumor initiation, malignant progression, and drug resistance [86, 87]. In other words, RNA therapy holds promise as an approach to “target the untargetable” and “treat the untreatable.”

To date, only one RNA-based therapeutic has been approved for cancer treatment. Imetelstat, a first-in-class telomerase inhibitor, received FDA approval in June 2024 and EMA approval in March 2025 for the treatment of adult patients with transfusion-dependent anemia due to very low-, low-, or intermediate-risk myelodysplastic syndromes (MDS) without isolated deletion 5q cytogenetic abnormality, who have had an unsatisfactory response to or are ineligible for erythropoietin-based therapy [88, 89]. Imetelstat is a 13-mer N3′–P5′ thio-phosphoramidate oligonucleotide that is linked to a palmitoyl lipid moiety via a 5′-thio-phosphate group. Its unique thio-phosphoramidate backbone provides enhanced stability, resistance to nuclease degradation, and the ability to form RNA duplexes. The lipid moiety facilitates cellular uptake and improves drug efficacy [90]. Unlike conventional ASOs, it primarily binds to the template region of the RNA component of human telomerase, preventing telomere binding. Telomerase activity and the expression of human telomerase reverse transcriptase (hTERT) RNA are significantly elevated in MDS and malignant stem and progenitor cells. Imetelstat treatment reduces telomere length, inhibits the proliferation of malignant stem and progenitor cells, and induces apoptotic cell death, ultimately leading to the reduction of malignant clones [89]. In a Phase III, randomized, double-blind, placebo-controlled, multicenter IMerge trial (MDS3001), imetelstat demonstrated its efficacy. It enabled patients to achieve sustained transfusion independence for approximately a year and showed activity in improving disease progression, with no serious adverse reactions observed [91].

Although no other ASO drugs have yet been approved for treating malignant tumors, the successful marketing authorization of imetelstat is representative of the feasibility of RNA-targeted therapies in oncology. It also highlights the value of conjugated oligonucleotide in overcoming the limitations of ASO. We believe this will drive momentum in the field and accelerate the progress of ASOs as therapeutic agents for oncology indications.

Numerous clinical trials are currently evaluating ASO drugs for various malignancies (Table 2), and we introduce here some examples of ASOs under development for targeting the “untargetable” in cancer.

**Table 2:** Representative clinical trials of ASO therapies in oncology

Drug	Target	Main indications/cancer types	Phase/current status	Reference
**Imetelstat (RYTELO, GRN163L)**	hTERT	Low-risk MDS, AML, myelofibrosis	FDA approved **Phase I/III**, recruiting	[88, 92, 93]
**Danvatirsen (AZD9150)**	**STAT3**	DLBCL, NSCLC, HNSCC	**Phase I/II**, completed Phase I/II**,** recruiting	[94–96]
**Oblimersen (G3139, Genasense)**	**BCL2**	CLL, AML, non-Hodgkin lymphoma	**Phase III**, completed	[97, 98]
**Custirsen (OGX-011)**	Clusterin	mCRPC, NSCLC, metastatic bladder cancer	**Phase III**, completed	[99, 100]
**Trabedersen (AP-12009,** OT-101**)**	**TGF-β2**	Glioma, pancreatic neoplasms, melanoma, colorectal carcinoma	Phase I/II, completed Phase II/III, recruiting	[101–103, 124]
**BP1001**	**GRB2**	AML, CML, solid tumors	**Phase I, completed Phase I/II,** recruiting	[104–106]
**LY2181308**	Survivin	AML, prostate cancer, NSCLC	**Phase II**, completed	[107–109]
**Apatorsen (OGX-427)**	**Hsp27**	Prostate, breast, bladder cancer, NSCLC	**Phase I/II**, completed	[110–113]
SD-101 (Nelitolimod)	TLR 9	Melanoma, HNSCC, pancreatic adenocarcinoma, NSCLC	**Phase I**/**II**, completed	[114–117]
**EZN-2968**	**HIF-1α**	Advanced solid tumors, lymphoma	**Phase I**, completed	[118, 119]
**AZD4785**	**KRAS**	NSCLC, advanced solid tumors	**Phase I**, completed	[120]
**GTI-2040**	**RNR R2**	Leukemia (AML, CML), advanced solid tumors, NSCLC	**Phase I/II**, completed	[121–123]

STAT3, Signal Transducer and Activator of Transcription 3; BCL2, B-Cell Lymphoma 2; TGF-β2, Transforming Growth Factor Beta 2; GRB2, Growth Factor Receptor-Bound Protein 2; Hsp27, Heat Shock Protein 27; TLR9, Toll-Like Receptor 9; HIF-1α, Hypoxia-Inducible Factor 1-alpha; KRAS, Kirsten Rat Sarcoma Viral Oncogene Homolog; RNR R2, Ribonucleotide Reductase Regulatory Subunit M2; MDS, myelodysplastic syndrome; AML, acute myeloid leukemia; DLBCL, diffuse large B-cell lymphoma; NSCLC, non–small cell lung cancer; HNSCC, head and neck squamous cell carcinoma; CLL, chronic lymphocytic leukemia; mCRPC, metastatic castration-resistant prostate cancer.

### Signal transducer and activator of transcription 3

The signal transducer and activator of transcription (STAT) proteins are a family of transcription factors with seven members: STAT1, STAT2, STAT3, STAT4, STAT5a, STAT5b, and STAT6 [125]. STAT3 is hyperactivated in most human cancers and is often associated with poor clinical prognosis [126]. STAT3 is typically activated by upstream growth factor kinases and cytokine receptors. Non-receptor tyrosine kinases can also cause constitutive activation of STAT3. Phosphorylated STAT3 translocates to the nucleus, where it transcribes and broadly regulates tumor-associated molecules such as vascular endothelial growth factor (VEGF), cyclin-dependent regulatory proteins D1 and D2 (Cyclin D1/D2), Vimentin, programmed death ligand-1 (PD-L1), p53, interleukin-12 (IL-12), and C-X-C motif chemokine ligand 10 (CXCL10), which leads to cellular angiogenesis, proliferation, metastasis, and immunosuppression [127, 128]. Based on the above, targeting STAT3 represents a promising therapeutic strategy for tumor treatment.

Danvatirsen (also known as AZD9150 or ISIS STAT3Rx) is a 16-nucleotide PS ASO with constrained ethyl nucleosides at 5′ and 3′ ends, which was designed to block STAT3 translation [129]. In a preclinical study, danvatirsen significantly reduced STAT3 expression and inhibited the tumor growth in lung and lymphoma xenograft models [130]. Danvatirsen was also demonstrated to induce hematopoietic differentiation in primary stem and progenitor cells from AML and MDS [131]. Based on these results, danvatirsen entered a Phase Ib clinical trial in patients with heavily pre-treated relapsed/refractory non-Hodgkin lymphomas [132]. Most enrolled patients (27/30) had diffuse large B-cell lymphoma (DLBCL) in which activated STAT3 is associated with poor overall survival [101]. Two complete and two partial responses were obtained among the patients with DLBCL, resulting in 13% of overall response rate. On the other hand, a shift in the profile of immune cells occurred in four patients. The authors indicate that danvatirsen is likely to have a positive immunomodulatory effect. Prioa *et al*. confirmed this point: in pre- and post-treatment biopsies from danvatirsen-treated patients, danvatirsen was observed to be selectively taken up by cells in the tumor microenvironment (TME) but not by cancer cells. Preclinical data showed that danvatirsen can reprogram the immunosuppressive TME, and when combined with anti-PD-L1 therapy, it enhanced antitumor activity [133].

In a Phase II clinical trial, durvalumab (a selective PD-L1 inhibitor) combined with danvatirsen treated 37 patients (29 pancreatic ductal adenocarcinoma (PDAC), 7 NSCLC, and 1 mismatch repair-deficient colorectal cancer), with no objective responses observed. However, four patients with NSCLC and four patients with PDAC exhibited a 4-month disease control rate (DCR). Preclinical evidence suggests that the lack of response may be due to danvatirsen-induced myeloid immune suppression [96]. In two other clinical trials of the combination of durvalumab and danvatirsen therapy for solid tumors, no significant objective responses were demonstrated [134, 135]. For hematologic malignancies, the combination of danvatirsen and acalabrutinib in relapsed or refractory DLBCL was reported to be safe and tolerable, but its antitumor efficacy was limited [136]. Two Phase I/II clinical trials are currently ongoing: one is evaluating danvatirsen in combination with pembrolizumab for the treatment of recurrent/metastatic head and neck squamous cell carcinoma (NCT05814666); the other is danvatirsen monotherapy followed by combination with venetoclax for patients with relapsed/refractory MDS or AML (NCT05986240).

### Transforming growth factor beta

Transforming growth factor-β (TGF-β) is a multi-functional cytokine that regulates multiple cellular processes. Particularly, TGF-β plays an important pathogenetic role in the late stage of tumor progression and therapeutic resistance [137]. Overexpression of TGF-β is associated with invasive behaviors in a variety of cancers, including glioma, pancreatic cancer, and colorectal cancer [138, 139].

Trabedersen (also known as AP-120009 and OT-101) is a PS ASO that induces RNase H–dependent degradation of TGFB2 mRNA (which encodes TGF-β) [139, 140]. In several *in vivo* preclinical studies, TGFB2 expression in cancer cells was significantly downregulated upon trabedersen treatment [101, 141]. In the following clinical trials, trabedersen has shown its tolerance and efficacy in treating gliomas [101, 102, 124]. Unfortunately, the Phase III clinical trial of trabedersen for glioma treatment was halted primarily due to insufficient patient numbers. Additionally, some have pointed out design flaws in the Phase I/II trial, resulting in the insufficient statistical power of the results [90]. In a Phase I/II pancreatic cancer trial, 37 advanced (Stage IVA or IVB) PDAC patients treated with trabedersen showed no severe adverse reactions, indicating overall safety. For patients on a 140-mg/m^2^/day dose (4-day-on/10-day-off schedule), the observed overall survival (OS) was 14.5 months, more than double the survival time with approved standard treatments in second-line settings. One patient with metastatic pancreatic cancer achieved long-term complete remission [142]. Another Phase II/III clinical trial (NCT06079346) began in 2024 to compare the efficacy and safety of trabedersen in combination with mFOLFIRINOX (folinic acid, 5-FU, irinotecan, oxaliplatin) versus mFOLFIRINOX alone in advanced and unresectable or metastatic pancreatic cancer patients. Trabedersen also has ongoing Phase I/II clinical trials in thoracic and respiratory malignancies. One trial aims to assess the safety and tolerability and the initial clinical efficacy of trabedersen in combination with pembrolizumab in patients with malignant pleural mesothelioma failing to respond to checkpoint inhibition (NCT05425576). Another trial evaluates the safety and efficacy of trabedersen in combination with pembrolizumab for the treatment of NSCLC (NCT06579196).

### Tumor protein 53

p53 (which is encoded by *TP53* gene) is “the guardian of the genome” and is the most mutated gene across cancers. Although ~40%–50% of cancers have genetic alterations in *TP53*, a specific drug that targets its functions and improves the therapeutic outcomes of cancer patients has not been widely used yet.

**Figure 2:**
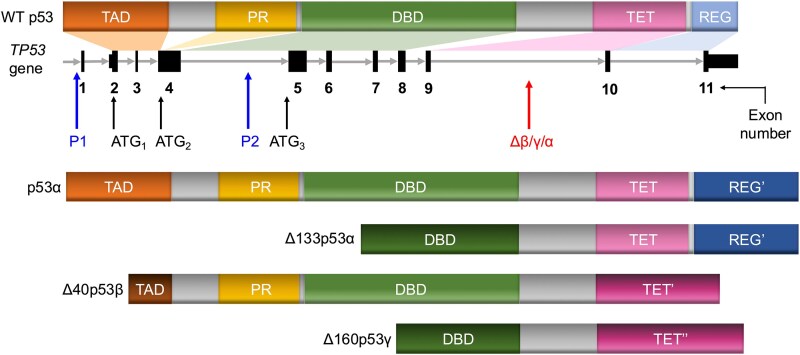
Schematic representation of the *TP53* gene, p53 protein, and its functional domains and isoforms. WT: wild-type, TAD: transactivation domain, PR: proline-rich domain, DBD: DNA-binding domain, TET: tetramerization domain, REG: regulatory domain. Four of the theoretical 12 isoforms are shown as examples.

The *TP53* gene uses alternative promoters, downstream initiation codons, or alternative splicing to express at least 12 protein isoforms (Fig. 2) [143–145]. More specifically, an alternative promoter (“P2”) in intron 4 yields “Δ133p53” isoforms that lack the transactivation domain (TAD) and a part of the DNA-binding domain (DBD). Other categories of p53 isoforms are generated by alternative ATG codons (codon 40 and codon 160), which activate translation initiation of “Δ40p53” and “Δ160p53,” respectively. Therefore, there can be four types of p53 isoforms with different N termini (wild-type (WT) p53, Δ133p53, Δ40p53, and Δ160p53). In addition, alternative splicing of intron 9 generates three isoforms with distinct C-terminal domains. Theoretically, this will result in the production of 12 p53 isoforms in total, each with a combination of differential N and C termini. Among these isoforms, Δ133p53 has been shown to possess oncogenic functions through its antagonistic effects against WT p53 [145–147]. Interestingly, several ASOs targeting the 5′-terminal region of P2 were designed based on the secondary structure model of Δ133p53 mRNA. Although the experiments were done with cells transfected with a plasmid encoding flag-tagged Δ133p53, expression of Δ133p53 was reduced by the ASO treatment in several cancer cell lines [147].

*Cenersen* is a 20mer PS ASO complementary to exon 10 of the p53 mRNA [148]. Cenersen was previously used in clinical trials for hematologic malignancies. A Phase II randomized study of cenersen plus idarubicin (± cytarabine) was performed in patients with relapsed/refractory AML. Results showed that 10 of the 53 (19%) patients responded (8 complete remission (CR) and 2 CR with incomplete platelet recovery) [149]. Studies have also been conducted in high-risk chronic lymphocytic leukemia: cenersen + FCR (fludarabine/cyclophosphamide/rituximab) in 20 patients. The overall response rate was 53% (CR 18%), but the sample size was small, and no control group was included [150]. Multiple early studies have shown that transient p53 inhibition can alleviate treatment-related toxicities in normal tissues during chemotherapy or radiotherapy, such as hair loss, gastrointestinal damage, and bone marrow suppression [151–154]. In clinical studies of cenersen, suppression of p53 function in combination with chemotherapy for AML did not result in increased severity or the emergence of specific toxicities compared with chemotherapy alone.

Given that mutations in *TP53* often involve both loss-of-function and oncogenic gain-of-function, future ASO design may focus on selective inhibition of mutant p53 gain-of-function activities, thereby increasing tumor cell sensitivity to DNA-damaging agents. The sequence specificity of ASOs also enables the precise targeting of distinct p53 isoforms, allowing more refined functional modulation within the complex biological context of p53. Owing to the complexity of its structure, function, and biological effects, p53 remains a challenging and largely “undruggable” target, and therapeutic strategies aimed at p53 continue to face substantial obstacles.

### Metastasis-associated lung adenocarcinoma transcript 1

*MALAT1* (metastasis-associated lung adenocarcinoma transcript 1, also known as *NEAT2*) is among the most abundant long ncRNAs (lncRNAs) whose aberrant expression has been investigated in numerous cancers [155, 156]. *MALAT1* is a highly conserved lncRNA that was first identified in lung cancers [157]. Since the first report, it is now recognized that *MALAT1* is highly expressed in >20 different solid or lymphoid tumors [158]. MALAT1 is a multi-functional lncRNA that regulates transcriptional elongation via association with elongation factors, RNA splicing through controlling localization and distribution of splicing factors, chromatin compaction via affecting histone modification, protein–protein interaction as a scaffold, and several signaling pathways through regulations on gene expression and translation [156, 159, 160].

Based on the pathological and clinical implications of *MALAT1*, several research groups have developed ASO-based therapeutics to inhibit MALAT1 lncRNA. Amodio *et al*. developed a novel LNA-gapmer ASO to knock down MALAT1 expression. The ASO induced apoptosis in both *in vitro* and *in vivo* cell line–derived xenograft (CDX) models of human multiple myeloma (MM) via downregulation of the two major activators of proteasome gene activation [161]. Adewunmi *et al*. utilized the triple-negative breast cancer (TNBC) mouse model to knock down MALAT1 RNA expression using ASO and observed delayed primary tumor growth, reduced cell proliferation, and enhanced apoptosis. In preclinical models, ASO combined with carboplatin or anti-PD-1 enhanced therapeutic response [162]. In another study, MALAT1-ASO treatment significantly inhibits the growth both *in vitro* and *in vivo* models [163]. FTX-001 is also an ASO targeting MALAT1 based on cEt chemical modification, whose potency and safety have been also demonstrated *in vivo* [164]. These studies have provided proof of concept that targeting lncRNAs with RNA therapeutics is a promising strategy for cancer treatment.

## Conclusions

As highlighted throughout this review, ASO-based RNA therapeutics provide a direct and versatile approach for targeting specific molecules. This precision expands therapeutic opportunities to previously “undruggable” targets and enables more rapid and cost-effective drug development compared with modalities such as antibody-based therapies or traditional chemotherapeutics [117]. Recent work even demonstrated that splice-switching ASOs can be used not only to silence genes but also to engineer new antitumor molecules—e.g. by inserting toxic exons into TRA2β transcripts to generate tumor-suppressive lncRNAs [165]. Such advances underscore the transformative potential of ASO-based strategies in cancer therapy.

Despite these promising developments, only one RNA therapeutic has been approved for oncology to date, reflecting significant challenges that must be overcome. Most currently approved ASOs are administered parenterally. Following systemic administration, ASOs undergo rapid tissue distribution, followed by a slower elimination phase, and chemical modifications have extended their half-lives from hours to weeks. At the cellular level, ASOs are taken up predominantly via endocytosis and, after endosomal/lysosomal trafficking, reach their intracellular sites of action, enabling sustained suppression of target RNAs. Nevertheless, *in vivo* degradation and limited intracellular uptake efficiency remain major challenges for their broader clinical application.

Even when ASOs successfully reach their intended molecular targets, tumor heterogeneity can substantially limit therapeutic efficacy. One fundamental barrier is that many cancers cannot be eradicated by targeting a single molecule. Achieving complete tumor control may require either identifying a critical dependency essential for tumor survival or simultaneously targeting multiple vulnerabilities. In line with the latter concept, dual-siRNA strategies—such as co-targeting VEGF and kinesin spindle protein (KSP)—have shown intriguing therapeutic potential [166]. Moreover, tumor cells may undergo adaptive changes during disease progression, including alterations in splicing patterns, target gene dependencies, and RNA stability, leading to therapeutic resistance. If these challenges can be overcome, ASO-based therapies may be most powerful when precisely tailored to each patient’s tumor genomic profile as part of precision oncology [167].

Another major obstacle lies in drug delivery. Although substantial efforts have been made to improve ASO stability, tissue specificity, and cellular uptake, additional breakthroughs in drug delivery systems (DDS) will be necessary for cancer-selective ASO therapeutics to achieve their full clinical impact. Liver-targeted delivery of ASOs has become relatively well established, whereas the development of effective extrahepatic delivery strategies remains a major goal in the field. With the rapid development of artificial intelligence (AI), the integration of AI and machine learning may further accelerate the identification of ASO sequences with high target specificity and reduced off-target potential, as well as the optimization of delivery vehicles such as lipid nanoparticles (LNPs), thereby enhancing the clinical feasibility of these agents.

In summary, continued integration of biological insight to identify actionable cancer dependencies, together with technological innovation to improve efficacy and delivery, will be critical for realizing the full potential of RNA therapeutics in cancer treatment.

## Abbreviations

**2′-cEt**, 2′-constrained ethyl; **2′-F**, 2′-fluoro; **2′-OMe**, 2′-*O*-methyl; **2′-O-MOE**, 2′-*O*-methoxyethyl; **AIDS**, acquired immunodeficiency syndrome; **ALL**, acute lymphoblastic leukemia; **AOC**, antibody–oligonucleotide conjugate; **ApoC-III**, apolipoprotein C-III; **ASO**, antisense oligonucleotide; **ATTR**, amyloidosis transthyretin; **BCL2**, B-cell lymphoma 2; **CDX**, cell line–derived xenograft; **cEt**, constrained ethyl; **CLL**, chronic lymphocytic leukemia; **CMV**, cytomegalovirus; **CR**, complete remission; **CML**, chronic myeloid leukemia; **CRPC/mCRPC**, metastatic castration-resistant prostate cancer; **CXCL10**, C-X-C motif chemokine ligand 10; **DBD**, DNA-binding domain; **DCR**, disease control rate; **DDS**, drug delivery system; **DLDCL**, diffuse large B-cell lymphoma; **DMD**, Duchenne muscular dystrophy; **DMPK**, dystrophia myotonica protein kinase; **DNA**, deoxyribonucleic acid; **EGFR**, epidermal growth factor receptor; **EMA**, European Medicines Agency; **ESE**, exonic splicing enhancer; **FDA**, Food and Drug Administration; **FCS**, familial chylomicronemia syndrome; **GalNAc**, *N*-acetylgalactosamine; **GRB2**, growth factor receptor-bound protein 2; **HAE**, hereditary angioedema; **HIF-1α**, hypoxia-inducible factor 1-alpha; **HNSCC**, head and neck squamous cell carcinoma; **hTERT**, human telomerase reverse transcriptase; **Hsp27**, heat shock protein 27; **HER2**, human epidermal growth factor receptor 2; **IE2**, immediate early gene 2; **IL-12**, interleukin-12; **ISS**, intronic splicing silencer; **KSP**, kinesin spindle protein; **KRAS**, Kirsten rat sarcoma virus oncogene homolog; **LNA**, locked nucleic acid; **lncRNA**, long non-coding RNA; **LNP**, lipid nanoparticle; **MALAT1**, metastasis-associated lung adenocarcinoma transcript 1; **MDS**, myelodysplastic syndromes; **MFSD8**, major facilitator superfamily domain-containing protein 8; **miRNA**, microRNA; **MM**, multiple myeloma; **mRNA**, messenger RNA; **NMD**, nonsense-mediated decay; **NGD**, no-go decay; **NSCLC**, non–small cell lung cancer; **OS**, overall survival; **PDAC**, pancreatic ductal adenocarcinoma; **PD-L1**, programmed death ligand-1; **PKK**, prekallikrein; **PNA**, peptide nucleic acid; **PMO**, phosphorodiamidate morpholino oligomer; **PR**, proline-rich domain; **PS**, phosphorothioate; **RNR R2 (RRM2)**, ribonucleotide reductase regulatory subunit M2; **RNA**, ribonucleic acid; **RNase H**, ribonuclease H; **RISC**, RNA-induced silencing complex; **siRNA**, small interfering RNA; **SMN1**, survival motor neuron 1; **STAT3**, signal transducer and activator of transcription 3; **TAD**, transactivation domain; **TET**, tetramerization domain; **TG**, triglyceride; **TGF-β2 (TGFB2)**, transforming growth factor beta 2; **TLR9**, toll-like receptor 9; **TME**, tumor microenvironment; **TNBC**, triple-negative breast cancer; **TTR**, transthyretin; **VLDL**, very-low-density lipoprotein; **VEGF**, vascular endothelial growth factor; **WT**, wild-type

## Data Availability

Not applicable.
